# A Pilot and Feasibility Study of Combined Oral Contraceptive Pills and Metabolic Outcomes in Premenopausal Women with Overweight or Obesity

**DOI:** 10.3390/women6010019

**Published:** 2026-03-04

**Authors:** Adnin Zaman, Aaron Lazorwitz, Myla Strawderman, Hong Hong Liu, Sarah A. Tydings, Susan W. Groth, Victoria A. Catenacci, Elizabeth A. Thomas

**Affiliations:** 1 Division of Endocrinology, Diabetes and Metabolism, Department of Medicine, University of Rochester Medical Center, Rochester, NY 14642, USA; 2 Division of Endocrinology, Metabolism and Diabetes, Department of Medicine, University of Colorado Anschutz Medical Center, Aurora, CO 80045, USA; 3 Anschutz Health and Wellness Center, University of Colorado Anschutz Medical Center, Aurora, CO 80045, USA; 4 Division of Complex Family Planning, Department of Obstetrics, Gynecology & Reproductive Sciences, Yale University, New Haven, CT 06510, USA; 5 Department of Biostatistics and Computational Biology, University of Rochester Medical Center, Rochester, NY 14642, USA; 6 University of Rochester School of Medicine and Dentistry, University of Rochester Medical Center, Rochester, NY 14642, USA; 7 School of Nursing, University of Rochester Medical Center, Rochester, NY 14642, USA; 8 Rocky Mountain Regional Veterans Affairs Hospital, Aurora, CO 80045, USA

**Keywords:** combined oral contraception, nonhormonal contraception, body weight, obesity, appetite, pilot, feasibility

## Abstract

Combined oral contraceptive pills (COCPs) are commonly used by reproductive-aged women with overweight or obesity, but their metabolic effects remain understudied. This pilot study examined the feasibility of recruiting and retaining women with overweight or obesity initiating COCPs and evaluated changes in body weight, body composition, energy intake (EI), eating behaviors, and cardiometabolic markers. Premenopausal women aged 18–40 years with a body mass index between 25 and 45 kg/m^2^ initiating COCPs (n = 10) or using nonhormonal contraception (NHC; n = 10) were followed for six months. Outcome measures included body weight, body composition, EI, eating behavior questionnaires, ecological momentary assessment of appetite and satiety, and fasting laboratory measures. There were no between-group differences in changes in weight, EI, or appetite. Binge-eating severity decreased in COCP users and increased in NHC users, though the within-group change in COCP users was not statistically significant. Exploratory analyses demonstrated increases in hemoglobin A1c and triglycerides among COCP users compared to NHC users, while bioavailable testosterone decreased in COCP users only. This study demonstrates high retention and feasibility among women with overweight/obesity undergoing intensive dietary and metabolic monitoring. Although weight outcomes were similar between groups, these preliminary findings identify potential metabolic signals warranting confirmation in adequately powered studies.

## 1. Introduction

More than 40% of reproductive-aged women in the United States (U.S.) have overweight or obesity (body mass index [BMI] ≥ 25 kg/m^2^), increasing their susceptibility to cardiometabolic disease [1]. Additionally, premenopausal women with overweight or obesity may gain weight at a faster rate during their reproductive years than those with a normal BMI (18.5–24.9 kg/m^2^) [2]. Since up to 80% of premenopausal women also use combined oral contraceptives (COCPs) [3], understanding whether COCPs accelerate this weight gain trajectory remains crucial, particularly in women already at elevated metabolic risk. Although past studies have reported minimal weight change with COCP use [4], these prior investigations relied on retrospective designs, short follow-up durations, or predominantly normal-weight cohorts, limiting generalizability to women with established overweight or obesity.

The hormonal milieu of COCP users differs substantially from that of normally cycling women. COCPs suppress endogenous ovarian hormone production while providing daily exposure to synthetic estrogens and progestins, resulting in altered sex-steroid patterns compared to natural cyclic fluctuations [5]. Experimental and clinical studies suggest that exogenous sex steroids may influence lipid metabolism, glucose regulation, appetite, and food-related reward processing [6–9]. Thus, there is biologic plausibility for potential changes in cardiometabolic markers or eating behaviors following COCP initiation. However, prospective studies evaluating changes in body weight, body composition, validated eating-behavior measures, and cardiometabolic risk factors specifically among women with overweight or obesity initiating COCPs remain limited.

Few prior studies have assessed the feasibility of recruiting and retaining women with overweight/obesity initiating COCPs for longitudinal follow-up with intensive weight and health-related outcome measures. In this prospective observational pilot and feasibility study, we assessed the ability to recruit and retain premenopausal women with overweight/obesity initiating COCPs or using nonhormonal contraception (NHC) over 6 months and compared changes in prespecified secondary outcomes—including body weight, body composition, energy intake (EI), eating behaviors, appetite, and markers of cardiometabolic health between groups.

## 2. Methods

This single-site, prospective observational pilot study recruited premenopausal women aged 18–40 years old with a BMI (calculated as weight in kilograms divided by height in meters squared) between 25 and 45 kg/m^2^ who were free of major medical or psychiatric illnesses. Women were excluded if they had metabolic or endocrine disorders (i.e., diabetes, polycystic ovary syndrome, congenital adrenal hyperplasia), a history of weight loss surgery, use of medications known to affect body weight (e.g., systemic glucocorticoids, stimulants, weight loss pharmacotherapy, metformin), current tobacco use, recent pregnancy-related events (i.e., current or planned pregnancy, abortion, miscarriage, or delivery), lactation, current or anticipated enrollment in a weight management program, or planned major dietary or physical activity changes during follow-up.

Participants were not randomized; contraceptive choice was determined prior to study enrollment. Women not using hormonal contraception who elected to start the norgestimate/ethinyl estradiol 0.25 mg/35 mcg COCP during a routine contraceptive counseling visit were enrolled in the COCP group. Baseline assessments were conducted prior to COCP initiation in this group. Women using NHCs (e.g., the copper intrauterine device, male condoms, tubal ligation, partner vasectomy, withdrawal, natural family planning, abstinence) who did not plan to start hormonal contraception were enrolled in the NHC group. Participants were recruited from the University of Colorado Anschutz Medical Campus (CU-AMC) and surrounding community via flyers and social media strategies.

Feasibility outcomes included recruitment, retention, and completion of primary study assessments. Recruitment was tracked through screening logs, eligibility determination, and consent. The recruitment rate was calculated as the proportion of eligible women who enrolled. Retention was defined as the proportion of enrolled participants who completed baseline and 6-month visits. Feasibility of data collection was assessed by the proportion of participants completing weight assessment at both timepoints.

Clinical outcome measures were obtained at baseline and at 6 months. Body weight was measured in the morning following an overnight fast using a calibrated digital scale with participants wearing only a hospital gown. Height was measured to the nearest 1 mm on a stadiometer at baseline to calculate BMI. Waist circumference and seated blood pressures were obtained using standard procedures. Body composition (fat mass and lean mass) was assessed with dual-energy X-ray absorptiometry (DXA; Hologic Horizon W, version Apex 5.6.04). Self-reported dietary energy and macronutrient intake were assessed with 3-day diet diaries analyzed using Nutrition Data System for Research (NDSR version 2019; University of Minnesota, Minneapolis, MN, USA), and Healthy Eating Index (HEI) scores to assess diet quality were derived from dietary data [10]. Pre- and post-meal hunger and satiety were measured using ecological momentary assessment before and after each meal for the three days coinciding with the 3-day diet records [11]. To assess eating behaviors, we administered the Perceived Stress Scale (PSS) [12]; Binge Eating Scale (BES) [13]; Palatable Eating Motives Scale (PEMS) [14]; version 2 of the reduced, 18-question Three-Factor Eating Questionnaire (TFEQ) [15]; Reward-Based Eating Drive Scale (RED) [16]; and Food Choices Inventory (FCI) [17]. Participants also underwent a 12 h fasted blood draw to measure glucose, insulin, hemoglobin A1c (HbA1c), lipids (total cholesterol, triglycerides, high density lipoprotein [HDL], low-density lipoprotein [LDL]), leptin, ghrelin, adiponectin, albumin, testosterone, and sex-hormone binding globulin (SHBG) levels. We calculated the homeostatic model assessment of insulin resistance (HOMA-IR) and serum bioavailable testosterone (BAT) from these measurements [18,19].

Participants completed interval questionnaires at 3 and 6 months to assess changes in health status, medication use, continued use of the same COCP formulation or NHC method(s), and self-reported changes in physical activity or major dietary patterns. This questionnaire was developed by the study team to monitor potential confounding changes during follow-up.

### Statistical Analysis:

Recruitment and retention rates of eligible participants were calculated with 90% two-sided Clopper–Pearson confidence intervals (CI). Descriptive statistics were used to summarize baseline and six-month measures, as well as changes over time. Within-group changes were evaluated using the Wilcoxon Signed Rank test. Between-group differences of the change across six months were described using the Hodges–Lehmann estimate (HLE), which provides the median of all possible pairwise group differences. The Wilcoxon Rank Sum test was used to compare the COCP and NHC groups across all measured outcomes. Statistical results of between-group changes are reported as the difference in change between COCP and NHC. As this was a pilot and feasibility study, statistical significance was set at α = 0.10 without adjustments for multiple comparisons. All analyses were conducted using SAS statistical software (v9.4, SAS Institute, Cary, NC, USA).

## 3. Results

**Figure 1.**
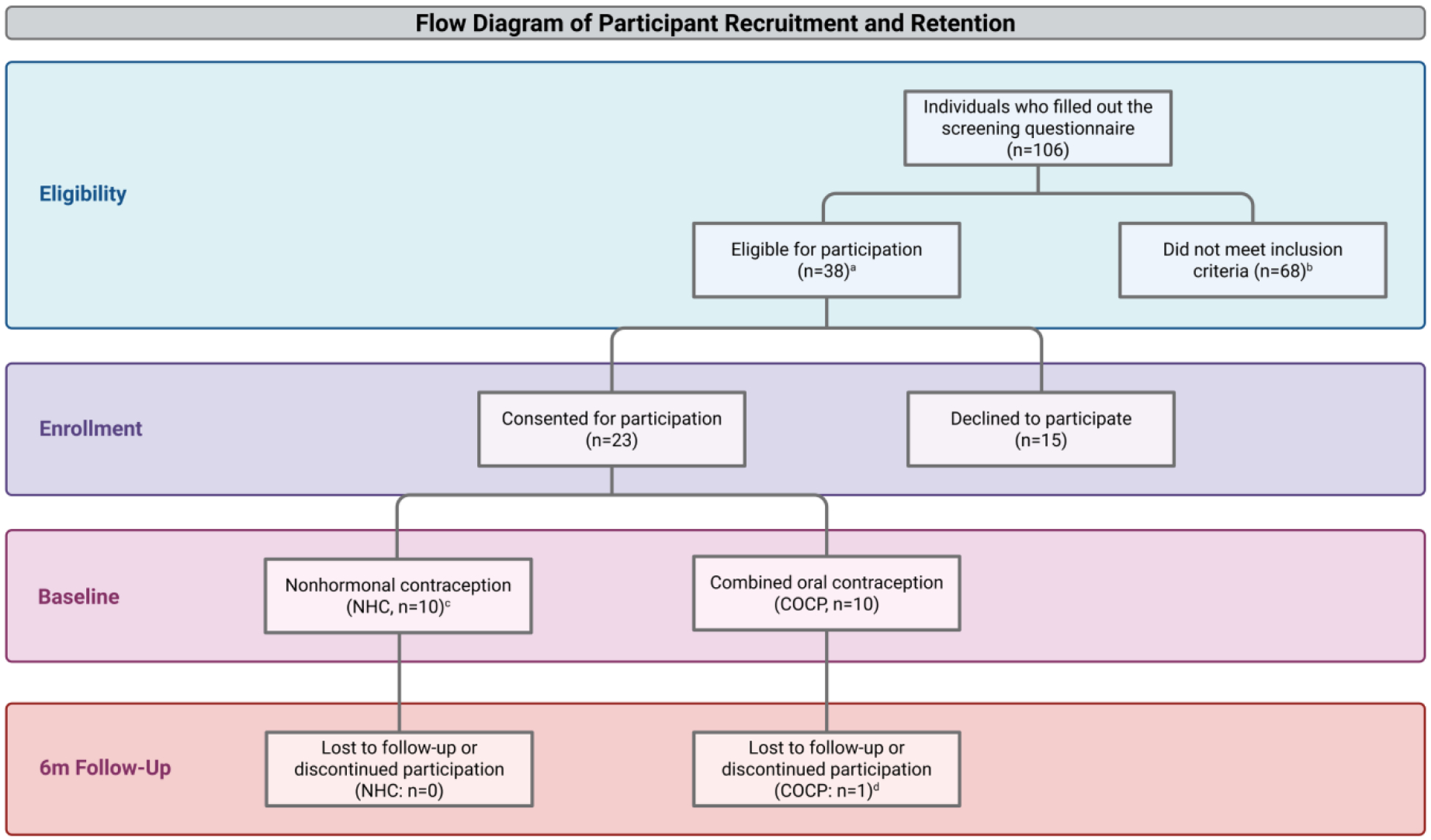
Flow diagram of participant recruitment and retention in premenopausal women with overweight/obesity using COCPs vs. NHCs at CU-AMC (November 2021–June 2023). Women were eligible for inclusion if they were 18–40 years old, had a BMI between 25 and 45 kg/m^2^, and were free of major medical or psychiatric illnesses. Women were excluded if they had metabolic or endocrine disorders (i.e., diabetes, polycystic ovary syndrome, congenital adrenal hyperplasia), a history of weight loss surgery, used medications affecting body weight (i.e., systemic glucocorticoids, stimulants, weight loss pharmacotherapy, metformin), current tobacco use, recent pregnancy-related events (i.e., current or planned pregnancy, abortion, miscarriage, or delivery), lactation, current or anticipated enrollment in a weight management program or research study with a dietary or physical activity component, or planned major dietary or physical activity changes. ^a^ Of the 38 women who were eligible for participation, 23 (61%) provided written consent. The remaining 15 women either did not respond to additional outreach attempts (n = 8), declined participation after screening (n = 6), or completed screening after the recruitment period ended (n = 1). ^b^ Among 68 women who were not eligible, the primary reasons for exclusion included BMI too low (n = 41), BMI too high (n = 1), current or recent (<3 m) hormonal contraception use (n = 19), breastfeeding (n = 1), weight loss surgery within the last year (n = 1), use of medications potentially influencing weight (n = 2), heavy smoking (n = 1), or current enrollment in a weight management program or research study (n = 2). ^c^ Three women who consented did not complete baseline measures and were lost to follow-up prior to data collection. Those who were not using hormonal contraceptives and elected to start the norgestimate/ethinyl estradiol 0.25 mg/35 mcg COCP after a contraceptive counseling visit were enrolled in the COCP group. Women who chose to continue using NHCs (i.e., the copper intrauterine device, male condoms, tubal ligation, partner vasectomy, withdrawal, natural family planning, abstinence, etc.) were included in the NHC group. ^d^ One participant did not complete 6-month measures in the COCP group and withdrew from the study due to worsening mental health after initiating the norgestimate/ethinyl estradiol 0.25 mg/35 mcg pill. Created in BioRender. Zaman, A. (2026) https://BioRender.com/a21jj54 (accessed on 27 February 2026).

Of the 106 women who completed the screening questionnaire, 38 (35.8%) were eligible for participation. Of these, 23 (60.5%; CI: 45.9–73.8%) provided consent and 20 (87.0%; 90% CI: 69.6–96.3%) completed baseline measures (COCP n = 10, NHC n = 10). Six-month completion rates were 90% for COCP users (1 participant withdrew prior to 6-month assessment) and 100% for NHC users (Figure 1). All retained participants completed primary anthropometric assessments at both timepoints, demonstrating feasibility of recruitment and longitudinal follow-up in this population. No participants reported initiating a structured weight management program or major changes in either dietary patterns or physical activity during follow-up.

The median (range) age of all participants was 29.6 (23.0–37.4) years. Eighty-five percent of women were White, and fifteen percent reported Hispanic ethnicity, with a higher proportion of Hispanic participants in the COCP group. Most participants (95%) had completed at least some college education. Baseline demographic, socioeconomic, reproductive history, and lifestyle characteristics were generally similar between groups (Table 1).

Anthropometric, metabolic, and behavioral measures at baseline were also comparable between groups. These parameters, along with 6-month changes in these secondary outcomes, are summarized in Table 2. There were no significant between-group differences in 6-month changes in body weight, BMI, waist circumference, total or fat mass, blood pressure, fasting glucose, insulin, or HOMA-IR. HbA1c decreased in NHC users (median Δ *−*0.1%) and increased in COCP users (median Δ +0.1%), resulting in a significant between-group difference (COCP vs. NHC HLE 0.2%; 90% CI: 0.1, 0.3). Similarly, triglycerides decreased in NHC users and increased in COCP users (HLE 53.5 mg/dL; 90% CI: 25.0, 84.0). BAT increased in NHC users and decreased substantially in COCP users (HLE *−*22.4%; 90% CI: *−*34.7, *−*15.5).

With respect to eating behaviors, binge eating severity (BES score) increased in NHC users (median Δ +3.5) and decreased in COCP users (median Δ *−*3.5), yielding a significant between-group difference (HLE *−*5.5; 90% CI *−*9.0, *−*2.0). Cognitive Restraint on the TFEQ—which measures conscious efforts to control or restrict food intake [20]—decreased within the COCP group but did not differ significantly between groups. Finally, NHC users had significantly greater improvements in HEI scores compared to COCP users (HLE *−*9.4; 90% CI *−*21.5, 0.4). No additional between-group differences were observed in changes in EI, other validated eating-behavior measures, ecological momentary ratings of pre-meal hunger and post-meal satiety, or remaining fasting laboratory parameters.

## 4. Discussion

This observational pilot and feasibility study demonstrates that it is possible to recruit and retain premenopausal women with overweight or obesity initiating COCPs alongside a comparison group using NHCs and longitudinally compare changes in body weight; body composition; and metabolic, dietary, and appetite-related outcomes. Recruitment and retention is a known challenge in contraceptive studies where women are often reluctant to be randomized to birth control methods [21]. However, our retention rates of 90% (COCP) and 100% (NHC) suggest that this observational approach may facilitate research in this area [4].

We observed no significant between-group differences in changes in body weight or body composition over six months of COCP versus NHC use. However, this pilot and feasibility study was not powered to detect differences in these parameters, and unadjusted results were likely influenced by residual confounding due to the small sample size. NHC users trended toward weight gain, while COCP users tended to lose weight, which coincides with NHC users experiencing greater binge eating severity. The literature suggests that exogenous sex steroids may influence appetite regulation and eating behavior through hormonal and neurobiological pathways [7,8]. As a pilot study with a small sample size and observational design, these findings should be interpreted as exploratory and hypothesis-generating. The observed differences may reflect true behavioral or physiologic adaptations but may also represent variability inherent to small samples or residual confounding.

Because participants self-selected their contraceptive method, unmeasured differences in motivation, weight-related attitudes, or health behaviors between groups may have contributed to observed differences in eating behaviors. Repeated assessment of dietary and behavioral measures may have also introduced reporting effects or assessment reactivity over time. Finally, outcome measures were not standardized to menstrual cycle phase in the NHC group. Fluctuations in endogenous estrogen and progesterone across the cycle may influence fluid balance, appetite, and eating behavior, potentially introducing variability in weight and metabolic assessments. In contrast, hormonal exposure in the COCP group was relatively stable. In a small sample, such differential variability may have attenuated or exaggerated between-group differences and, therefore, represents an important limitation of this design.

Dietary quality (HEI) also differed between groups at follow-up; however, this reflected an improvement in NHC users rather than a decline in COCP users. A similar pattern was seen for Cognitive Restraint on the TFEQ [15]. Although Cognitive Restraint decreased in COCP users, the magnitude of change was comparable in NHC users, and the between-group difference was not significant. As with other secondary outcomes in this pilot study, these findings warrant replication in larger, adequately powered investigations.

COCP users exhibited increases in HbA1c and triglycerides, while NHC users showed decreases in these parameters. Although prior studies have reported alterations in lipid profiles and glucose metabolism with hormonal contraceptive use [6,9,22], the present findings should be interpreted cautiously, given the pilot design and limited sample size. The observed decrease in BAT is biologically plausible given estrogen-induced increases in SHBG [23], though this too requires confirmation. These laboratory shifts may reflect pharmacologic effects of COCP use; however, causal inference cannot be drawn from the current observational design. Observed changes in eating behavior measures further underscore the need for adequately powered studies to disentangle behavioral and physiological contributors to weight regulation and energy balance in this population.

While prior studies examining the association between COCP initiation and weight changes have been inconclusive, they have predominantly excluded individuals with higher BMIs [4]—the focus of our study. It remains plausible that women with overweight or obesity are at greater risk of contraceptive-associated weight gain given their propensity to gain weight more rapidly over their lifetimes than their normal-weight counterparts [2], and that exogenous sex steroids in COCPs may influence energy balance through multiple mechanisms [8]. This concern is also particularly important, as the perception of weight gain with hormonal contraceptive use may influence their uptake in women with overweight/obesity [24].

## 5. Conclusions

This pilot and feasibility study demonstrates that it is possible to recruit and retain women with overweight/obesity to investigate the effect of cOCPs initiation on body weight, body composition, and cardiometabolic health. However, larger studies powered to detect changes in weight and cardiometabolic parameters—along with concurrent exploration of underlying mechanisms—are needed, as this question remains clinically important for women with overweight/obesity who have largely been overlooked in prior research. This pilot study provides both a framework and preliminary data to serve as a steppingstone for the design of such future investigations.

## Figures and Tables

**Table 1.** Baseline characteristics in premenopausal women with overweight/obesity using COCPs vs. NHCs.

Baseline Characteristics			
Characteristic	All Participants (N = 20)	NHC (n = 10)	COCP (n = 10)
Age (yrs)			
*Median (IQR)*	29.6 (27.1, 33.6)	30.3 (27.3, 34.6)	29.0 (25.0, 33.0)
Race			
*White*	17 (85.0)	8 (80.0)	9 (90.0)
*Asian*	2 (10.0)	2 (20.0)	0 (0.0)
*Other*	1 (5.0)	0 (0.0)	1 (10.0)
Ethnicity			
*Non-Hispanic*	17 (85.0)	10 (100.0)	7 (70.0)
*Hispanic*	3 (15.0)	0 (0.0)	3 (30.0)
Highest Level of School			
*12th Grade*	1 (5.0)	0 (0.0)	1 (10.0)
*2-year College*	1 (5.0)	0 (0.0)	1 (10.0)
*4-year College*	9 (45.0)	5 (50.0)	4 (40.0)
*Master’s Degree*	7 (35.0)	5 (50.0)	2 (20.0)
*Doctorate Degree*	1 (5.0)	0 (0.0)	1 (10.0)
*Other*	1 (5.0)	0 (0.0)	1 (10.0)
Annual Household Income			
<*$25,000*	1 (5.0)	0 (0.0)	1 (10.0)
*$25,001-$45,000*	0 (0.0)	0 (0.0)	0 (0.0)
*$45,001-$70,000*	11 (55.0)	5 (50.0)	6 (60.0)
*$70,001-$110,000*	5 (25.0)	4 (40.0)	1 (10.0)
>*$110,001*	3 (15.0)	1 (10.0)	2 (20.0)
Marital Status			
*Single*	8 (40.0)	5 (50.0)	3 (30.0)
*Committed Relation*	8 (40.0)	3 (30.0)	5 (50.0)
*Married*	4 (20.0)	2 (20.0)	2 (20.0)
Age at Menarche (yrs)			
*Median (IQR)*	12 (11, 13)	12 (10,13)	11.5 (11,13)
Normal Periods in Last Year?			
*% Reported Yes*	16 (80.0)	8 (80.0)	8 (80.0)
Ever Pregnant?			
*% Reported Yes*	3 (15.0)	0 (0.0)	3 (30.0)
Overweight as a Child?			
*% Reported Yes*	13 (65.0)	6 (60.0)	7 (70.0)
Highest Reported Adult Weight (kg)			
*Median (IQR)*	85.3 (81.9, 92.8)	87.3 (79.4, 93.0)	83.9 (83.9, 90.7)
Currently Exercising?			
*% Reported Yes*	15 (75.0)	9 (90.0)	6 (60.0)
Currently Using…			
*Tobacco (% Yes)*	0 (0.0)	0 (0.0)	0 (0.0)
*Alcohol (% Yes)*	17 (85.0)	8 (80.0)	9 (0.0)
*Recreational Drugs (% Yes)*	9 (45.0)	4 (40.0)	5 (50.0)

The baseline demographic and socioeconomic characteristics of the N = 20 women who enrolled and began the study are listed as n (%) unless otherwise specified.

**Table 2.** Secondary outcome measure results from baseline to 6 months in premenopausal women with overweight/obesity using COCPs vs. NHCs at CU-AMC (November 2021–June 2023).

**Variable**	**Baseline**		**6 Months**		**Within-Group Change** ^a^		**Between-Group Change** ^b^
	**NHC** (n = 10)	**COCP** (n = 10)	**NHC** (n = 10)	**COCP** (n = 9)	**NHC** (n = 10)	**COCP** (n = 9)	**COCP-NHC** (n = 19)
	*Median* *(IQR)*				*Median* Δ*, m6-0* *(IQR)*		*Median* Δ*, HLE* *(90% CI)*
*Body Weight*							
**Weight (lbs)**	185.1 (163.3, 198.5)	186.3 (175.8, 194.2)	187.7 (167.3, 198.9)	182.5 (180.9, 184.6)	2.9 (0.3, 3.6)	−3.3 (−4.4, −1.1)	−4.8 (−8.0, 3.2)
**BMI (kg/m^2^)**	30.5 (28.6, 33.0)	30.1 (28.9, 33.0)	30.8 (28.8, 33.4)	29.3 (28.6, 31.1)	0.4 (−0.2, 0.7)	−0.6 (−1.0, −0.4)	−0.8 (−1.5, 1.0)
*Body Composition (DXA)* ^c^							
**Total Mass (kg)**	83.0 (73.0, 88.7)	83.4 (78.4, 86.6)	84.1 (74.6, 92.2)	81.4 (81.1, 83.2)	1.0 (−0.4, 1.5)	−1.4 (−1.6, −0.8)	−2.1 (−4.5, 2.3)
**Fat Mass (kg)**	34.1 (28.8, 37.6)	36.3 (34.5, 37.5)	36.6 (30.0, 39.2)	36.2 (33.2, 36.7)	1.1 (0.1, 1.6)	−0.5 (−0.8, 1.9)	−1.0 (−3.2, 1.2)
**Lean Mass (kg)**	46.8 (43.5, 48.8)	46.0 (44.0, 48.2)	46.1 (44.0, 48.3)	45.2 (42.0, 47.0)	−0.3 (−0.7, 0.5)	−0.7 * (−1.4, −0.4)	−0.7 (−1.7, 0.2)
**%Body Fat**	41.3 (38.4, 44.6)	42.5 (41.1, 43.8)	43.1 (40.7, 44.9)	44.4 (41.0, 45.1)	1.0 ** (0.3, 1.1)	0.4 (−0.1, 1.8)	−0.5 (−1.8, 0.9)
*Blood Pressure* ^d^							
**SBP (mmHg)**	116.5 (113.5, 118.0)	117.5 (109.5, 122.5)	116.3 (112.5, 121.0)	114.0 (112.0, 119.0)	−0.5 (−4.5, 3.0)	1.0 (−4.0, 4.5)	1.0 (−5.5, 7.5)
**DBP (mmHg)**	75.0 (72.5, 78.5)	74.5 (70.0, 78.0)	72.5 (68.5, 76.0)	76.5 (70.0, 79.0)	−1.0 (−3.0, 1.0)	4.5 (−1.0, 6.0)	4.5 (−1.0, 7.5)
*Fasting Labs* ^e^							
**Glucose (mg/dL)**	86.5 (86.0, 91.0)	85.0 (83.0, 89.0)	89.0 (87.0, 90.0)	87.0 (86.0, 89.0)	1.5 (0, 3.0)	1.0 (−1.0, 3.0)	−1.0 (−5.0, 3.0)
**Insulin (uIU/mL)**	5.5 (4.0, 15.0)	6.5 (4.0, 14.0)	9.0 (5.0, 15.0)	9.0 (6.0, 13.0)	0.5 (0, 4.0)	2.0 ** (1.0, 2.0)	1.0 (−2.0, 3.0)
**HOMA-IR** ^f^	1.2 (0.9, 2.8)	1.4 (0.9, 2.9)	1.9 (1.1, 3.2)	2.0 (1.3, 2.6)	0.3 (−0.1, 0.9)	0.3 ** (0.1, 0.5)	0.06 (−0.45, 0.54)
**HbA1c (%)**	5.4 (5.3, 5.6)	5.3 (5.1, 5.4)	5.2 (5.2, 5.4)	5.4 (5.2, 5.6)	−0.1 ** (−0.2, 0)	0.1 (0, 0.1)	**0.2** †† **(0.1, 0.3)**
**Cholesterol (mg/dL)**	177.0 (159.0, 197.0)	153.0 (148.0, 158.0)	170.0 (152.0, 183.0)	150.0 (127.0, 178.0)	−13.0 (−22.0, 8.0)	0 (−17.0, 29.0)	11.5 (−12.0, 37.0)
**Triglycerides (mg/dL)**	112.0 (61.0, 168.0)	70.5 (58.0, 98.0)	109.0 (69.0, 129.0)	112.0 (88.0, 124.0)	−7.5 (−42.0, 13.0)	43.0 *** (26.0, 53.0)	**53.5** ††† **(25.0, 84.0)**
**HDL (mg/dL)**	54.0 (48.0, 58.0)	52.0 (45.0, 61.0)	49.5 (48.0, 52.0)	55.0 (42.0, 60.0)	−2.0 (−3.0, 1.0)	−1.0 (−5.0, 7.0)	1.0 (−6.0, 9.0)
**LDL (mg/dL)**	104.3 (76.4, 112.6)	90.3 (68.6, 96.4)	92.3 (80.8, 106.6)	77.8 (68.0, 86.4)	−7.0 (−20.6, −0.6)	−10.0 (−20.4, 16.8)	−1.8 (−19.8, 17.4)
**BAT**^g^ **(%)**	28.3 (26.9, 35.9)	30.9 (21.9, 44.7)	34.8 (31.5, 38.2)	12.4 (9.6, 13.6)	3.2 *** (2.7, 6.3)	−18.4 *** (−29.5, −12.3)	−**22.4** ††† **(−34.7, −15.5)**
*Validated Questionnaires* ^h^							
**BES Total**	12.0 (6.0, 15.0)	13.0 (7.0, 17.0)	13.5 (8.0, 21.0)	10.0 (7.0, 12.0)	3.5 ** (0, 6.0)	−3.0 (−5.0, 1.0)	−**5.5** †† **(−9.0, −2.0)**
**PEMS Total**	44.0 (41.0, 47.0)	34.0 (27.0, 38.0)	47.5 (35.0, 54.0)	34.0 (30.0, 41.0)	3.5 (−11.0, 12.0)	−3.0 (−5.0, 8.0)	−1.5 (−13.0, 10.0)
**PSS Total**	18.5 (11.0, 21.0)	17.5 (13.0, 22.0)	21.0 (15.0, 23.0)	18.0 (15.0, 20.0)	5.5 (−1.0, 8.0)	−2.0 (−3.0, 2.0)	−5.0 (−9.0, 2.0)
**TFEQ - UE**	37.0 (29.6, 40.7)	37.0 (11.1, 48.1)	42.6 (33.3, 59.3)	25.9 (14.8, 44.4)	5.6 (−7.4, 11.1)	0 (−3.7, 0)	−3.7 (−11.1, 3.7)
**TFEQ - CR**	38.9 (27.8, 50.0)	41.7 (38.9, 61.1)	36.1 (27.8, 38.9)	38.9 (33.3, 44.4)	−5.6 (−11.1, 16.7)	−5.6 * (−16.7, −5.6)	−8.3 (−27.8, 5.6)
**TFEQ - EE**	72.2 (55.6, 77.8)	44.4 (33.3, 55.6)	55.6 (44.4, 77.8)	33.3 (22.2, 33.3)	−11.1 (−22.2, 11.1)	−11.1 (−22.2, 0)	0 (−22.2, 11.1)
**RED Total**	20.5 (16.0, 26.0)	11.5 (6.0, 27.0)	21.5 (19.0, 31.0)	12.0 (10.0, 18.0)	0.5 (−3.0, 7.0)	1.0 (−3.0, 4.0)	0 (−6.0, 5.0)
**FCI Total**	7.8 (6.9, 10.5)	8.9 (7.5, 10.3)	8.3 (7.6, 9.6)	7.8 (6.8, 8.9)	−0.1 (−0.9, 1.3)	−0.5 (−1.3, 0.3)	−0.4 (−1.9, 1.0)
**High Fats**	1.4 (1.0, 1.8)	1.6 (1.1, 2.3)	1.4 (1.1, 1.9)	1.5 (1.1, 1.8)	0 (−0.4, 0.3)	0 (−0.3, 0)	0 (−0.3, 0.4)
**Sweets**	2.5 (1.5, 3.3)	2.1 (1.6, 2.4)	2.7 (2.3, 3.4)	2.0 (1.9, 2.3)	0.1 (−0.1, 0.4)	0 (−0.3, 0.3)	−0.13 (−0.63, 0.25)
**Carbs**	1.6 (1.0, 2.4)	2.3 (1.6, 2.9)	1.9 (1.4, 2.0)	1.6 (1.4, 1.9)	0.1 (−0.4, 0.4)	−0.3 (−0.4, 0)	−0.3 (−0.8, 0.1)
**Fast Food**	2.8 (2.3, 3.0)	3.0 (2.3, 3.5)	2.5 (2.0, 3.0)	2.8 (2.5, 2.8)	−0.1 (−0.5, 0.3)	−0.3 (−0.5, 0)	0 (−0.3, 0.3)
*Ecological Momentary Assessment of Appetite* ^i^ *: Hunger, and Satiety*							
**Pre-Meal Hunger**	57.7 (48.2, 60.2)	54.7 (46.8, 60.9)	62.5 (57.3, 69.8)	55.6 (52.9, 59.8)	5.6 * (−2.1, 11.0)	2.2 (−5.3, 5.8)	−4.4 (−13.0, 3.6)
**Pre-Meal Desire to Eat**	60.4 (55.2, 63.6)	55.8 (46.0, 63.8)	66.6 (47.9, 76.5)	58.5 (52.6, 61.7)	5.1 (−6.7, 8.9)	1.6 (−2.1, 12.7)	−1.5 (−9.1, 8.8)
**Pre-Meal Amount Could Eat**	52.9 (48.3, 61.5)	49.1 (44.0, 60.7)	60.9 (50.1, 73.4)	55.4 (50.1, 59.0)	4.8 (−2.6, 12.8)	5.8 (−1.2, 7.8)	−0.9 (−9.8, 8.0)
**Post-Meal Fullness**	61.4 (55.7, 67.6)	62.2 (58.1, 68.6)	69.1 (59.8, 76.1)	64.4 (55.5, 75.2)	7.3 * (0.4, 11.5)	−0.9 (−4.0, 2.6)	−7.0 (−12.4, 0.4)
*3-Day Diet Diaries* ^j^							
**Total EI (kcal)**	2221 (1970, 2568)	1761 (1481, 1899)	2087 (1697, 2297)	1611 (1404, 2044)	−186 (−575, 65)	−239 (−323, 280)	150 (−302, 620)
**Total EI (g)**	1848 (1455, 2282)	1781 (1332, 2197)	1800 (1485, 2006)	1494 (1351, 1768)	32 (−119, 25)	−92 (−190, 225)	−44 (−413, 513)
**Total Fat (g)**	109 (97, 119)	74 (66, 83)	84 (75, 101)	68 (53, 75)	−20 (−34, −2)	−5 ** (−28, −1)	6.8 (−24.7, 26.5)
**%Cal from Fat**	40.6 (38.4, 43.6)	38.4 (36.1, 39.8)	38.5 (35.7, 41.6)	34.7 (27.9, 36.2)	−4.3 (−7.9, 2.4)	−5.1 (−13.1, 0.7)	−1.6 (−9.2, 4.6)
**Total Carb (g)**	226 (177, 308)	178 (167, 208)	227 (158, 267)	202 (176, 234)	−8 (−83, 45)	19 (−4, 34)	30 (−27, 96)
**%Cal from Carb**	42.3 (34.7, 47.4)	43.3 (38.3, 47.1)	44.7 (37.0, 47.3)	45.8 (43.5, 47.0)	−2.0 (−4.7, 8.9)	1.4 (−1.6, 8.7)	4.5 (−4.4, 11.5)
**Total Protein (g)**	100 (73, 112)	69 (61, 108)	76 (71, 101)	68.9 (61.7, 81.7)	−4 (−34, 10)	8 (−26, 13)	3.3 (−15.6, 27.1)
**%Cal from Protein**	15.5 (13.8, 16.7)	16.7 (15.1, 18.5)	15.3 (12.9, 19.0)	16.9 (13.8, 19.8)	−0.4 (−1.4, 2.2)	−3.3 (−4.4, −0.3)	−2.4 (−5.3, 2.8)
**HEI Total**	51.5 (40.1, 60.7)	52.2 (46.7, 56.3)	56.9 (50.8, 64.3)	48.0 (46.2, 58.5)	10.2 ** (2.4, 15.4)	0.6 (−6.8, 5.8)	−**9.4** † **(−21.5, 0.4)**

**Abbreviations:** HLE = Hodges–Lehmann Estimate, BMI = Body Mass Index, DXA = Dual-Energy X-Ray Absorptiometry, SBP = Systolic Blood Pressure, DBP = Diastolic Blood Pressure, HOMA-IR = Homeostasis Model Assessment of Insulin Resistance, A1c = Hemoglobin A1c, HDL = High Density Lipoprotein, LDL = Low Density Lipoprotein, BAT = Bioavailable Testosterone, BES = Binge Eating Scale, PEMS = Palatable Eating Motives Scale, PSS = Perceived Stress Scale, TFEQ = Three-Factor Eating Questionnaire, UE = Unrestrained Eating, CR = Cognitive Restraint, EE = Emotional Eating, RED = Reward-Based Eating Drive, FCI = Food Choices Inventory, Carbs = Carbohydrates, EI = Energy Intake, HEI = Healthy Eating Index.

a Within-group change was assessed using the Wilcoxon Signed Rank test. Significance is indicated with * (≤0.10), ** (≤0.05), *** (≤0.01).

b Hodges–Lehmann estimate is the median of all possible pairwise differences between COCP and NHC observations of the change from baseline to 6 months. Between-group difference in change was assessed using the Wilcoxon Rank Sum test. Significance is indicated with † (≤0.10), †† (≤0.05), ††† (≤0.01).

c Body composition was measured via DXA (Hologic Horizon W, Apex Version 5.6.05 [Hologic, Inc.]).

d SBP and DBP were measured with a digital sphygmomanometer and reported as the average of two seated values.

e Labs were conducted on blood samples collected after a 12 h fast and run at the University of Colorado Clinical Translational Research Center.

f HOMA-IR was calculated as [insulin (mIU/mL) × glucose (mg/dL) × 0.55]/22.5.

g BAT% was calculated with the Vermeulen equation: BAT = [Testosterone]/[1 + (K_SHBG_ × SHBG) + (K_Albumin_ × Albumin)], where the association constants K_SHBG_ = 1.0 × 10^9^ L/mol and K_Albumin_ = 3.6 × 10^4^ L/mol.

h The BES was designed to assess the severity of binge eating behaviors by evaluating both the behavioral and emotional/cognitive aspects of binge-eating, with higher scores indicating greater binge eating severity. The PEMS assesses the reasons individuals eat palatable foods, with scoring reflecting the extent to which various emotional, social, and habitual motives influence eating behavior. The PSS aims to measure the perception of stress in an individual’s life, with scoring reflecting the degree to which situations are appraised as stressful over a specified period. The TFEQ assesses three distinct factors related to eating behavior—cognitive restraint (the conscious efforts to control or restrict food intake), disinhibition (the tendency to overeat in response to external cues or emotions), and hunger (the degree to which an individual experiences feelings of physical hunger and how much those feelings influence their eating behavior)—providing a score for each to understand how they influence eating patterns. The RED scale evaluates the drive to eat based on the rewarding properties of food, with scoring reflecting an individual’s sensitivity to food rewards and how it affects their eating behavior. Finally, the FCI measures the factors influencing food choices, with scoring reflecting the relative importance of various motivators such as health, taste, and convenience in food decision-making.

i Pre-meal hunger and post-meal satiety scores represent composite ecological momentary assessment (EMA) ratings averaged across breakfasts, lunches, and dinners over 3 days at each timepoint. Participants received EMA prompts via text before and after each meal, using a 0–100 sliding scale anchored by opposing descriptors: (1) *How hungry do you feel?* (0 = I am not hungry at all; 100 = I have never been more hungry), (2) *How strong is your desire to eat?* (0 = Very weak; 100 = Very strong), (3) *How much food do you think you could eat right now?* (0 = Nothing at all; 100 = Large amount), and (4) *How full do you feel?* (0 = I am not at all full; 100 = I have never felt more full).

j Dietary and macronutrient intake values are the composite scores from the self-reported 3-day diet diaries at each timepoint. Diet diaries were analyzed using Nutrition Data System for Research Software (version 2019, University of Minnesota).

## Data Availability

The data that support the findings of this study are openly available in ClinicalTrials.gov at https://clinicaltrials.gov/study/NCT05061472 (accessed on 27 February 2026), reference number NCT05061472. Individual participant data are not publicly available due to privacy and ethical restrictions. However, de-identified data may be made available upon reasonable request to the corresponding author, subject to Institutional Review Board (IRB) approval and completion of a data use agreement.
